# Analytical applications of smartphones for agricultural soil analysis

**DOI:** 10.1007/s00216-023-04558-1

**Published:** 2023-02-15

**Authors:** Marek Tobiszewski, Christina Vakh

**Affiliations:** Department of Analytical Chemistry, Chemical Faculty and EcoTech Center, Gdańsk University of Technology (GUT), Ul. G. Narutowicza 11/12, 80-233 Gdańsk, Poland

**Keywords:** Smartphone, Chemical analysis, Soil quality, Spectrophotometry, RGB model, Agriculture

## Abstract

Soil is one of the most important farming resources. Appropriate managing of its quality promotes productive and sustainable agriculture. The valuable farm practice in soil quality managing is based on regular soil analysis with the aim of determining the exact amount of nutrients or other chemical, physical, and biological soil properties. Soil analysis usually requires sample collection at the desired sampling depth followed by sample delivery to chemical laboratories. However, laboratory analyses are resource-intensive and costly, and require a lot of time, effort, and equipment. A low-cost, fast, and effective alternative for soil quality control is the application of smartphones to perform chemical analyses directly in the field or on the farm. In this paper, an overview of recent developments on smartphone-based methodologies for agricultural purposes and portable evaluation of soil quality and its properties is presented. The discussion focuses on recent applications of smartphone-based devices for the determination of basic soil parameters, content of organic matter, mineral fertilizers, and organic or inorganic pollutants. Obvious advantages of using smartphones, such as convenience and simplicity of use, and the main shortcomings, such as relatively poor precision of the results obtained, are also discussed. The general trend shows the huge interest from researchers to move the technology into the field with the aim of providing cost-effective and rapid soil analysis. This paper can broaden the understanding of using smartphones for chemical analysis of soil samples, as it is a relatively new area and is expected to be developed rapidly.

**Graphical Abstract:**

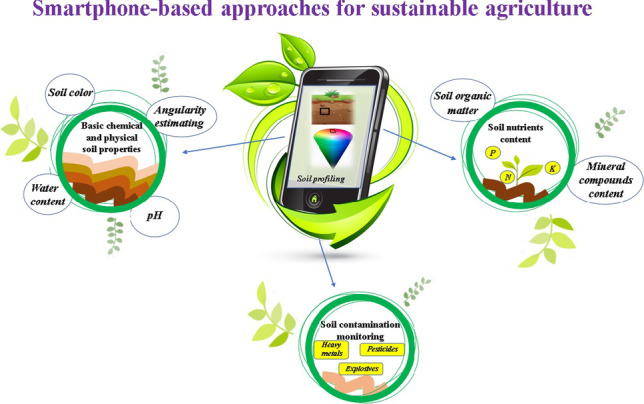

## Introduction

Chemical analysis of soil is a cost-effective management tool aimed at determination of nutrients (the content of elements, i.e. nitrogen, phosphorus, or potassium), chemical and physical soil properties (pH, cation exchange capacity, organic matter, soil texture), and chemical contaminants (e.g. heavy metals or persistent organic pollutants). Based on the laboratory measurements, decisions could be made about the species cultivated on a given soil, appropriate agrotechnical treatments (liming, fertilization), remediation processes, or abandoning cultivation in the case of high concentrations of pollutants [1]. In general, soil analysis usually requires sampling at desired sampling depth at a certain sampling site following by samples delivering to the analytical laboratories and analysis according to the appropriate procedure. However, soil analysis may appear to be problematic due to:

- *Potentially large distances from the sampling sites to the analytical laboratory,*
- *Large time delay between taking a sample and obtaining analytical measurement result,*
- *High costs of outsourced analyses.*

In order to overcome the above-mentioned problems, on-site chemical analysis can be performed directly in the sampling site without involving analytical laboratory. For this purpose, it is necessary to have an ease-of-operation, portable, and inexpensive measurement device. One of the possible ways in simplification of chemical analysis procedures is an application of computer scanners or cameras integrated with mobile phones instead of traditionally used skill-intensive laboratory equipment. The smartphone has been recognized as a powerful, inexpensive, and ease-of-use analytical platform for the colorimetric measurement of a variety of substances [2] which could be easily applied for *on site* analysis.

Agriculture has changed a lot in the past hundred years which is also reflected in the way how the soil quality control is performed. Base on the literature overview, it was found that the interest to smartphone application for soil analysis increases gradually and covers all possible tasks in managing of soil quality. On the one hand, such interest relates to the necessity to make soil quality control less tedious based on application of user-friendly approaches that are intuitive for farmers; on the other hand, smartphones application for soil testing could be a significant beneficence to farmers in developing countries without access to laboratories.

Several reviews have been presented to describe the application of smartphones in agriculture. The topics that have been discussed are monitoring of agri-food and water quality; seeds phenotyping; soil classifications [3]; farming and farm management which involve various day-to-day activities on the field, such as sowing, weeding, fertilizing, and making related agricultural decisions [4] including crop operations related to crop protection and diagnosis, nutrition and fertilization, and crop harvest [5]; and monitoring of operating behaviour of agricultural machinery cooperatives [6].

In comparison to previously reported reviews, this contribution provides the potential of smartphones application in soil chemical analysis and highlighted the importance of appropriate managing of soil quality and health for promotion of productive and sustainable agriculture. Developed solutions for determination of the content of soil basic parameters, nutrients, and environmental contaminants are discussed. The main benefits and shortcomings of smartphone-based procedures are presented through the application examples.

## Application of a smartphone as a detector

Nowadays, smartphones are increasingly considered as a portable and easy-used detection device, which could be applied for diagnostic, prognostic, quantification, or monitoring [7]. They are actively being used in many research fields including:

- Medicine as diagnostic tools at the point-of-care testing as well as noninvasive monitoring of disease conditions [8],
- Environment as devices for contamination monitoring and quality control of water [9], soil [10], or air [11],
- Food as a tool for products quality assessment [12, 13],
- Cultural heritage for in situ diagnosis of cultural heritage surfaces and objects [14],
- Agriculture for fast soil quality management, crop disease diagnosis [15], agri-food safety analysis [16].

**Fig. 1:**
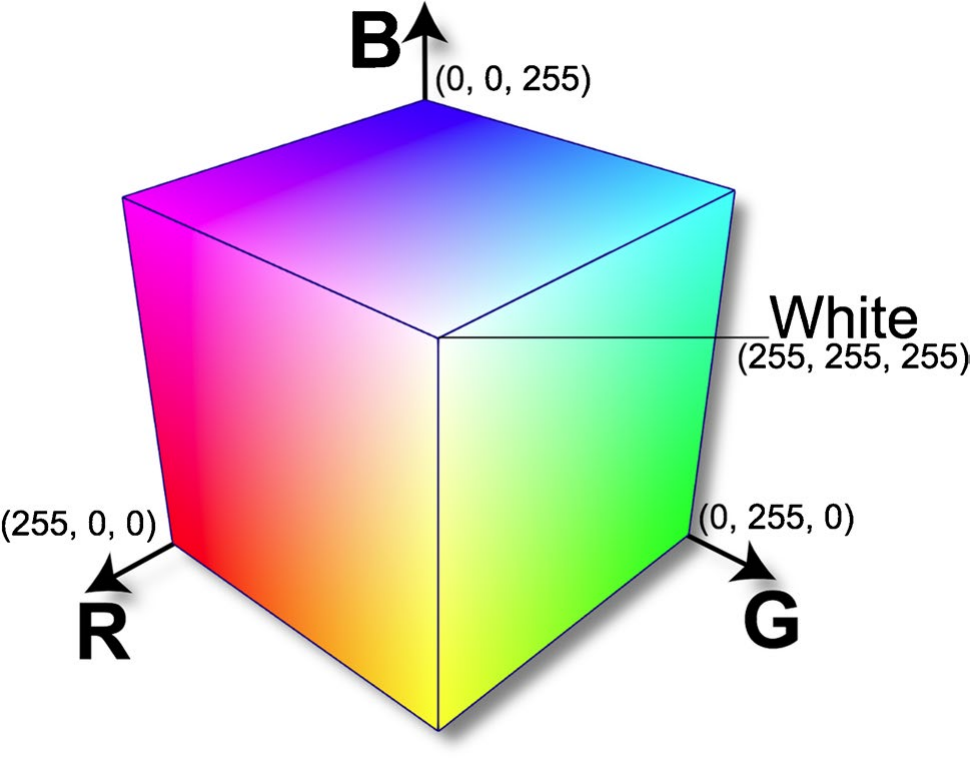
RGB colour space, placed in a three-dimensional coordinate system

Different kinds of sensors commonly used in smartphone could be applied for data acquisition. A standard smartphone is equipped with a number of built-in hardware sensors including image sensor (camera), audio sensor (microphone), an accelerometer, gyroscope, magnetometer, GPS, fingerprint identity sensor, proximity sensor, and light sensor, as well as sensors to collect information on ambient pressure, humidity, and temperature [17]. For example, accelerometer and gyroscope sensors were utilized for activity recognition of the user in daily life and have potential applications in healthcare systems [18]. Additionally, the mobile sensing application was developed for the android platform to gather GPS, acceleration, and microphone data from the mobile phone carried by a farmer while performing any activity for classification of a particular activity [19]. A novel ambient light sensor-based acetylcholinesterase colorimetric dipstick reader has been proposed for rapid organophosphate pesticides monitoring [20]. A magnetometer sensor has been utilized for robust structural health monitoring capable of identifying and evaluating different structural damage types [21]. However, for chemical analysis, camera image sensor is the most frequently used and assumes images acquisition and their digitizing [22]. Generally, detection is made after the smartphone-based device has been calibrated, using the calibration curve method for one-colour scale or with the use of artificial neural networks. The simplest way to use smartphones in the viewpoint of its application in chemical analysis is its combination with digital image colorimetry, where the proper colour spaces should be firstly selected according to different demand. The colour space for computerized display systems is often visualized using a three-dimensional coordinate system. Each colour (red, green, and blue) is assigned to one of the three orthogonal coordinate axes in three-dimensional space [22]. An example of a cube created in this way is shown in Fig. 1. Along each axis of the cube, the range of colours from the lack of this component to full colour saturation is presented. Any point (representing a colour) in a cube is defined by three numbers assigned to R (red), G (green), and B (blue), respectively. The diagonal line of the cube represents the colours from black (0, 0, 0) to white (1, 1, 1) through the shades of grey, and the three axes represent the colours red, green, and blue, respectively. In practice, the software will express the colours in the range 0–256 for each component. This RGB colour space is in the human perceptual space, which means that the RGB system represents fewer colours than the human can see.

## Assessment of basic chemical and physical soil properties

### Soil colour

**Fig. 2:**
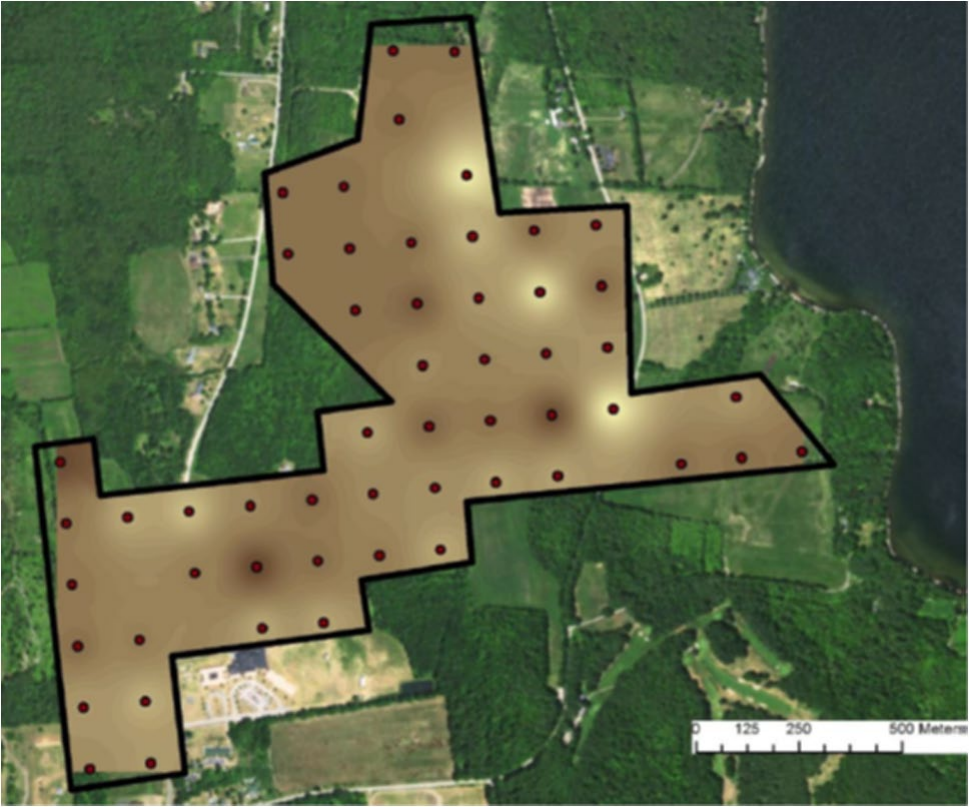
Sample soil colour map, created in a geographic information system based on the results of colour analyses obtained with a smartphone [24], reprinted with permission from Elsevier

Soil colour is one of the important properties used for classification and identification of soil strata. Depend on the colour, it is possible to conclude about the soil chemical composition, fertility, and water content. Attempts have been made to apply smartphone camera for the measurement of the soil colour. Various smartphone cameras were tested in the field study under bright sunlight and under overcast conditions. The results obtained were then compared with those of the visual assessment using Munsell soil colour cards. Additionally, spectrophotometry has been utilized as a reference method to confirm the results obtained with smartphone-based procedure. Soil colour determination results from smartphone measurements in both sunny and cloudy conditions were shown to be similar as those obtained with Munsell soil colour cards. However, the accuracy of measurements is influenced by lighting conditions, more accurate (more in line with the results obtained using the reference method) and more precise (characterized by lower variance) results were obtained for sunny conditions. The proposed mobile device has a great potential to enable users with no experience and lack access to colour charts to determinate colour of the soil [23]. The smartphone used to determine the colour of the soil allows for easy, automatic export of the results of determinations to an application based on geographic information systems [24]. Maps of this type, such as the one shown in Fig. 2, can be used to assess soil erosion, soil fertility, or water content.

**Fig. 3:**
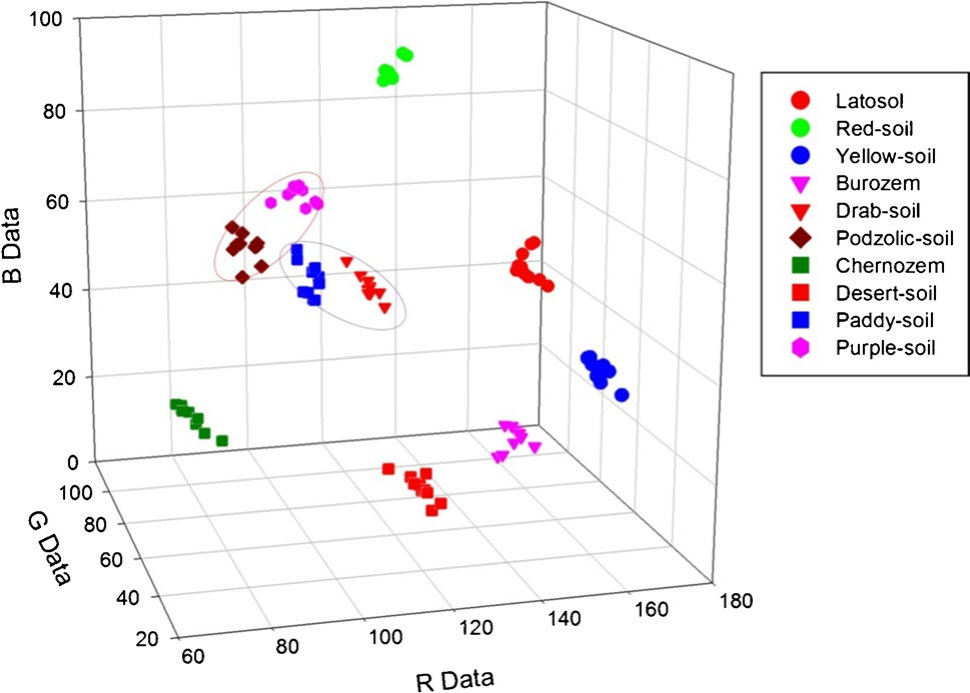
Classification of soil samples collected in China in the space expressed by the RGB model [25] with permission from Elsevier

The colour recognition system with a smartphone camera was developed to classify typical soils for agricultural land in China [25]. Photos of previously collected and air-dried soil samples were taken in a dark room, from a distance of half a meter, and in addition to the smartphone, the measuring set includes optical lenses, diaphragms, covers, and calibration cards. Outer lenses are used to adjust the size of the field of view. The shading device consists of a cover and cards for colour calibration, it is designed to work in conditions with no external light. The cover is made of a black plastic tube and prevents reflections. Figure 3 shows the results of classification of soil samples in the three-dimensional space R, G, and B. The algorithm was not able to clearly distinguish samples with loops-podzolic soils and purple soils as well as paddy soils and drab soils. Besides these problems, it was possible to classify the soil on the basis of the measurements of the colour of the soil.

**Fig. 4:**
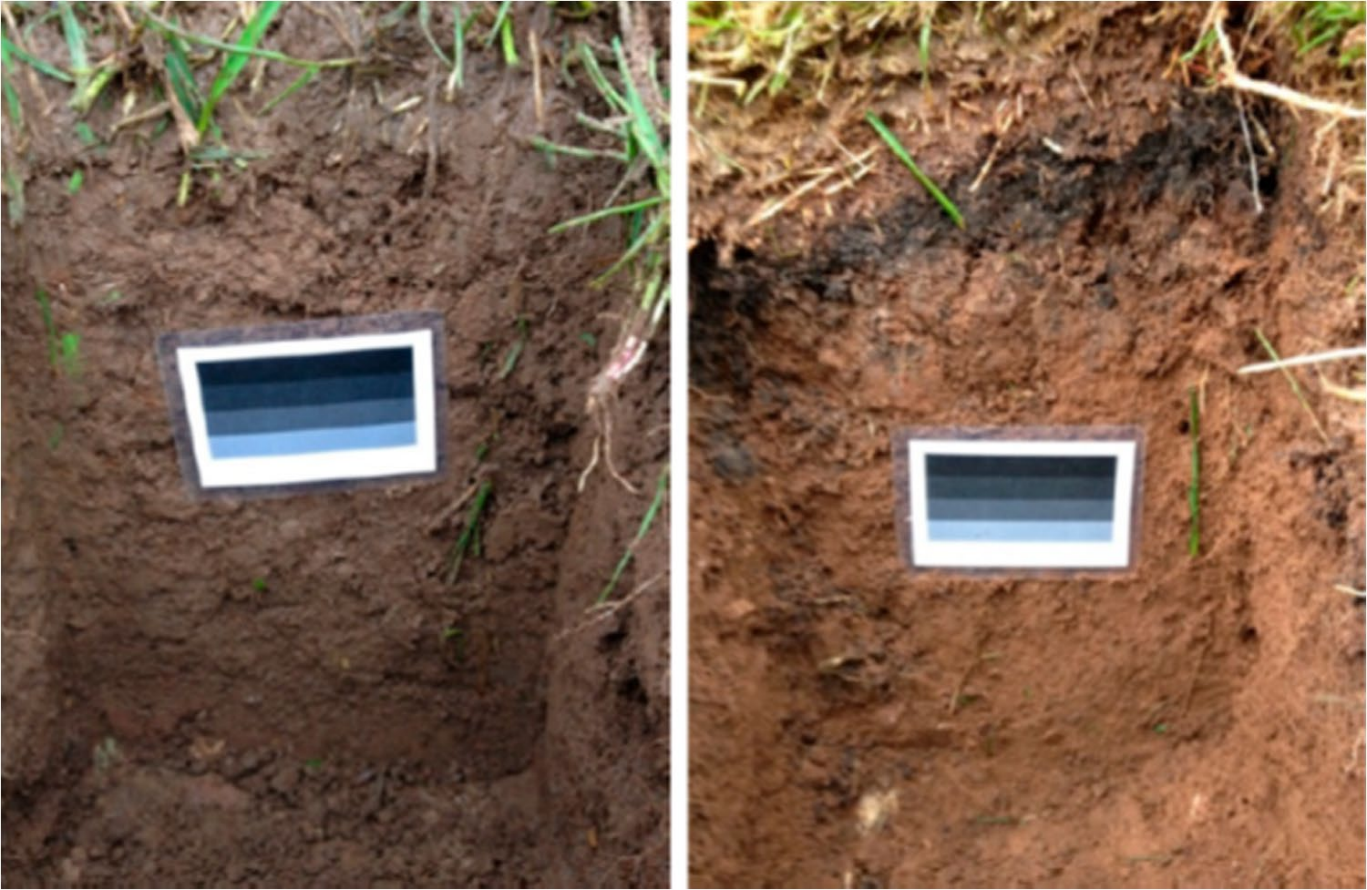
Photos of soil profiles with colour templates [26]

Another investigation aimed to determine soil structural parameters based on two groups of input data [26]. The first group includes the parameters obtained on the basis of photos from the RGB model, and the second one — the parameters obtained on the basis of the geographic location system and databases such as terrain, annual average rainfall and temperature, type of parent rock, or land cover with vegetation. Photographs were taken of soil profiles as shown in Fig. 4, using colour standards to compensate the influence of external conditions.

**Fig. 5:**
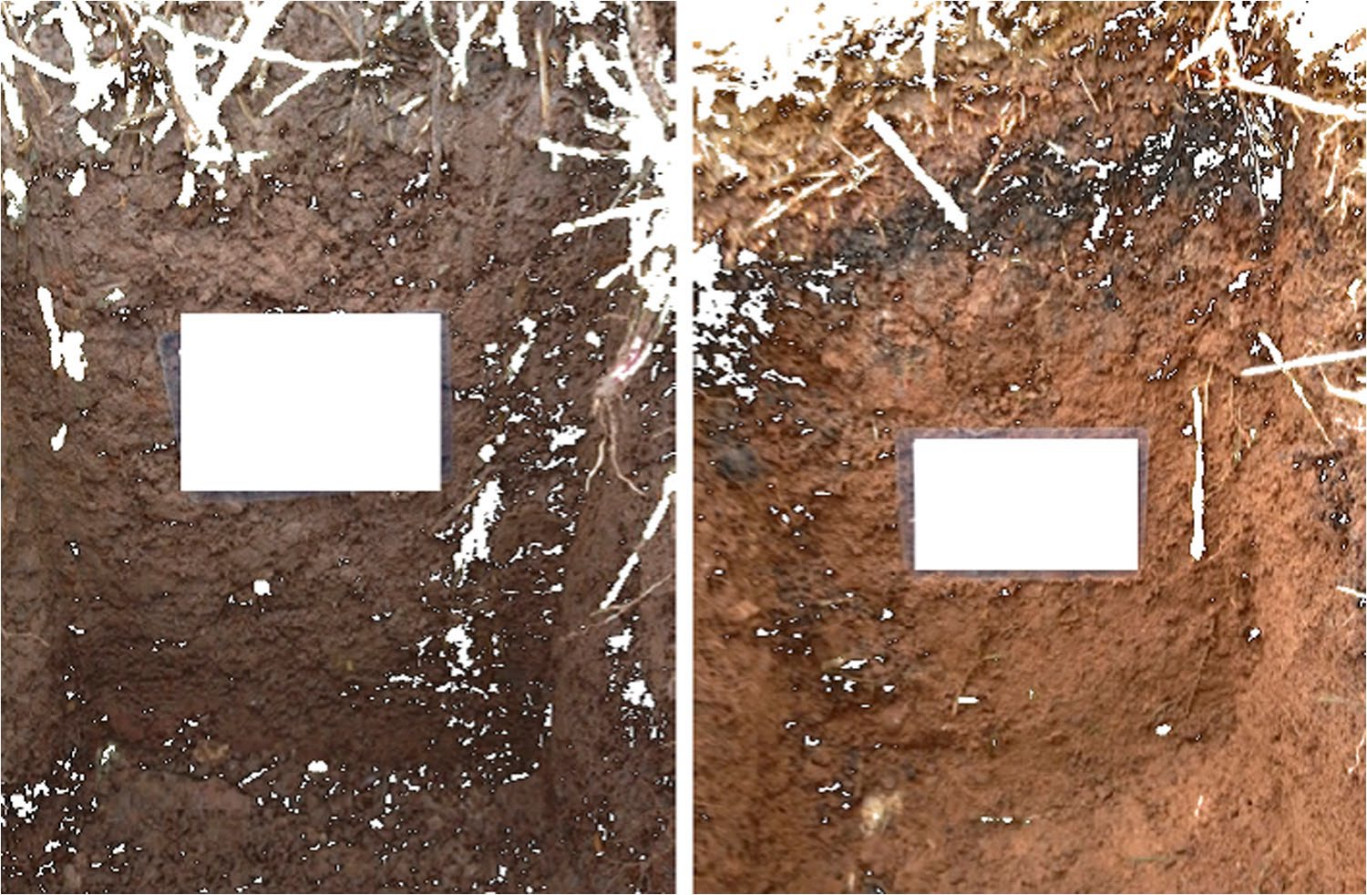
Photos of soil profiles, after removing elements not subject to analysis [26]

In order to properly analyse the colour of the soil profile photograph, all objects that may influence the analysis results and are not related to the soil colour should be removed from the picture. These are colour patterns themselves, above-ground and underground plant parts, and pieces of wood or stones. Figure 5 shows the modified photos of soil profiles. Using the data from the photos and location parameters, decent coefficients of determination were obtained for predicting the values of individual parameters — soil structure (*R*^2^ = 0.59), mass loss after burning as a measure of organic matter content (*R*^2^ = 0.64), drainage class (*R*^2^ = 0.50), density (*R*^2^ = 0.57), sand content (*R*^2^ = 0.58), dust content (*R*^2^ = 0.63), clay content (*R*^2^ = 0.62), and pH (*R*^2^ = 0.61). Prediction models based only on location parameters or based only on image parameters were also tested but such models gave worse prediction results. The continuation of research work consisted mainly in extending the range of estimated parameters with the content of elements — Al, B, Cu, Fe, Mn, Mo, P, S, Zn, Ca, Mg, Na. The ability of such models to predict the parameter values was much lower than in the previous studies (*R*^2^ coefficients ranging from 0.204 to 0.5) [27]. This may be due to the fact that the data for the localization parameters come from Ethiopian databases and in the first study came from Scottish databases.

### Measurement of pH

Among the soil properties, determination of the pH of the soil is highly important due to the acidity of the soil has an influence into the soil structure as well as solubility or absorption of nutrients. In agricultural practice, the pH of the soil is measured in order to determine the necessity of liming and to select a proper crop for cultivation. The soil pH value could be predicted by using an artificial neural network. Such a model should be previously trained to recognize images, based on a set of photos of soil samples with known pH values. The values of the linear regression coefficients for the results of determinations carried out on real samples in relation to the reference value are 0.823–0.859 for various features of the image. However, the investigation was done within a fairly narrow range of pH values between 7 and 8.5 [28].

Another approach assumes the application of smartphone at the pH reading stage with the utilization of classical indicators. Three indicators were tested: bromothymol blue, methyl red, and phenol red in the pH range between 1 and 13. The calibration curve of each indicator was prepared by determining the relationship between the pH values and the corresponding values of the RGB model [29]. The pH values are determined in a solution that has previously been in contact with the soil sample, which is actually a classic approach. The human factor when reading the colour is eliminated; therefore, more precise values of the determination results should be expected. Other studies show the possibility of using a universal indicator, which is a mixture of water, 1-propanol, phenolphthalein, sodium hydroxide, methyl red, bromothymol blue, sodium bisulfite, and thymol blue (and is usually applied to indicator paper). The procedure allows to determine of the pH precisely in the solution after contact with the soil in the range 4–9. As part of the solution, an add-on for spectrophotometric measurements under constant conditions (without the influence of external light) was printed in 3D printing technology [30].

### Water content measurement

The water content is determined in order to assess the air–water ratios and select the appropriate plant for cultivation or to provide correct watering. The water content of the soil sample can also be determined from photographs. For this purpose, 150 soil samples were analysed in order to determine the water content, with simultaneous photos taken under controlled conditions. Twenty-two variables, which are predictors, were read. The training dataset prepared in this way was used to find the relationship between the predictors and the water content in soil samples. Several models were used, while better results were obtained from the model based on artificial neural networks (determination coefficient *R*^2^ = 0.91) [31]. None of the predictors alone correlated well with water content, but the use of several of them to train the network gave satisfactory results.

### Angularity estimating

Particle angularity significantly affects the macro-mechanical behaviour of granular soils. However, the characterization of the particle angularity is rather difficult; thus, this fundamental soil property is commonly ignored by researchers. Nevertheless, the laboratory-on-a-smartphone device has been proposed for automatic evaluation of the particle angularities of soils. Machine learning techniques, including speed up robust features, k-means, and support vector machine, were used to train a soil image classifier [32]. After, soil image classifier automatically analyses the sharpness of particle corners in three-dimensional soil assembly images and classifies images based on Powers’ chart with a high classification accuracy of 93%.

## Determination of the soil nutrients content

### Measurement of carbon content

In general, the soil carbon content is an approximation of the humic substances content. Humic substances affect water retention, balance of soil minerals, improve root growth conditions, and reduce soil mechanical resistance. Attempts have been done to develop analytical procedures for the determination of carbon or organic matter in soil samples with the use of smartphone devices. The smartphone with a custom-made extension was used to take photos of 90 samples collected from three different types of soil. To compensate illumination varying effect, the illumination factor component of the image has been removed. Based on the photos received, it is possible to predict the content of organic matter with reasonable accuracy (*R*^2^ = 0.88) using the original images after minor modification [33]. The relationship between the RGB model parameters and the content of organic matter was found. Another investigation compared the effectiveness of predicting soil organic carbon from soil colour using a smartphone. Soil colour measurements were performed for samples with organic carbon content ranging from 0.03 to 4.74%. Photos of the samples were taken under standardized conditions. Various models and colour space descriptors were tested for predicting soil organic carbon.

Modelling was performed using multiple linear regression and a random forest method. Better prediction results were obtained on the basis of the colour analysis of moist soils and the organic carbon content was well correlated with the parameters describing the brightness of the colours. Both models under optimal conditions gave similar prediction efficiency with *R*^2^ values of 0.66 and 0.63. The use of a smartphone camera was characterized by a very similar accuracy of the obtained measurement results as in the case of a professional camera [34].

It is also important to establish the impact of the smartphone model, which camera is used for determinations, on the results of soil organic carbon content. For this purpose, the five most popular phones were compared based on how often they were purchased in the first half of 2020. SAMSUNG Galaxy Note10 + 5G, HUAWEI Mate 30 Pro, APPLE iPhone 11, OPPO Reno3 Pro 5G, and XIAOMI 10 Pro were compared, all sold on market from year 2019 to 2020. All smartphones gave comparable results of colour readings, and the values of the coefficients of determination of the calibration models were in the range of *R*^2^ = 0.79–0.82, which is important from the point of view of the availability and universality of the proposed software algorithms [35]. In the summary, the authors define several ways to make the software used more universal, compatible with a wide range of smartphone models.

### Measurements of mineral compounds content

Mineral soil composition is a fundamental feature that affects the properties and functions of soil [36]. Additionally, minerals are necessary for plant growth, their deficits lead to plants low growing as well as pathological changes in leaves. In agricultural practice, the measurement of the mineral compounds content is required for correct fertilization process and setting the doses of fertilizers. A smartphone-based procedure was developed for the fast and easy-operated determination of soil mineral compounds content.

A procedure was developed based on application of Android smartphones in combination with commercially available Quantofix® test strips which were impregnated with impregnation reagents. In this case, smartphone is used as a portable analytical device that reads the colours from the test strips, which are correlated with the concentrations of the individual minerals present in the soil. The smartphone results were compared to those obtained by standard methods for the determination of extractable N-nitrogen (nitrates) and phosphorus (phosphates). Determination of the nitrate content is carried out after standard sample preparation, which assumes extraction of the target analytes with deionized water. The procedure is applicable in the concentration range up to 100 mg L^−1^ NO_3_^−^. For the phosphates determination, a mixture of 0.05 N HCl and 0.025 N H_2_SO_4_ has been utilized. In both cases, colour-forming reaction has been proceed in the test strips. However, the determination of the phosphate content was hampered by the influence of interfering substances present in the samples. The procedure can be used as a screening tool to assess the concentration of minerals in the soil. Three different smartphones were used in the study, from different price ranges. The results obtained using a smartphone from the “top shelf” were characterized by the best precision and accuracy, and worse cameras gave results with considerable systematic error [1].

The continuation of research on the use of smartphones to determine the content of nitrates and phosphates in soil where vegetables were grown allowed to evaluate the practical aspects of using such a tool [10]. The approach is primarily a convenient way to optimize the soil fertilization process. Significant financial savings can be achieved by abandoning the use of fertilizers in situations where the N and P content in the soil is already sufficient or even exceeds the needs of crops. Such information can help to improve the efficiency of the use of mineral fertilizers in small farms, reduce fertilization costs, and reduce the risk of nitrate and phosphate leaching into the aquatic environment. Determination of N and P in soil could be the first step to introducing a better crop nutrient management model by applying nutrient corrections and will help to reduce the continued, uncontrolled use of mineral fertilizers. This, in turn, may contribute to reduction of the eutrophication process of surface waters, reduction of soil acidification, or the risk associated with the presence of heavy metals in soil.

**Fig. 6:**
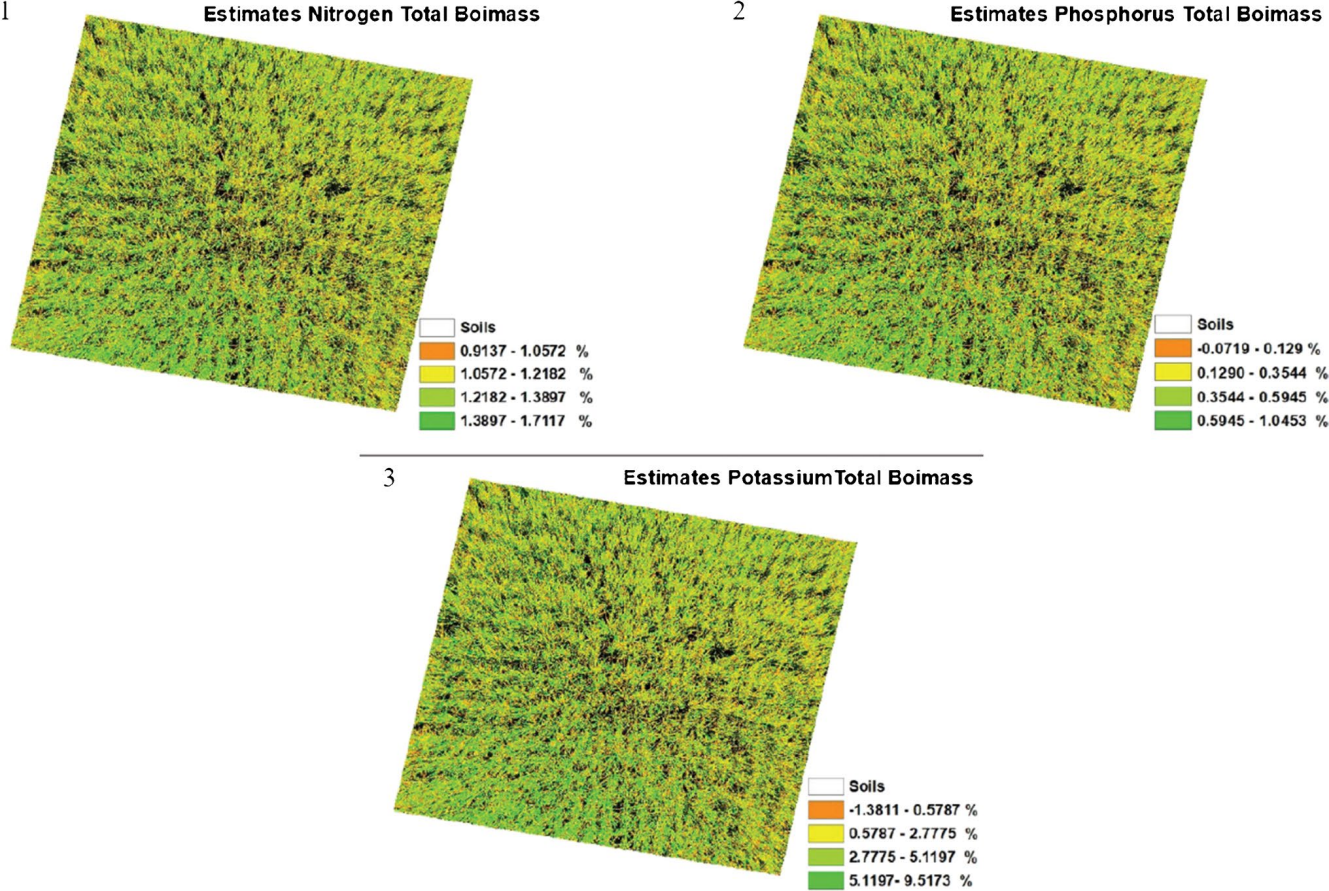
Estimation results of the concentration of (1) nitrogen, (2) phosphorus, and (3) potassium in rice biomass. The photos were taken from 5 m with a smartphone camera [37]

An interesting analytical approach is taking photos of plant crops and based on the colours of the crops, determination whether there is a sufficient concentration of mineral fertilizers in the soil. This approach was used to assess the content of nitrogen, phosphorus, and potassium in biomass, particularly during the cultivation of rice [37]. The measurement is based on the taking a photo of the crop from a height of 5 m using a long selfie stick, which allows to capture larger, more representative area. Figure 6 shows examples of this type of photos. Calibration was performed with the use of concentration data determined using classical analytical procedures. The calibration curves are the relationships between the concentrations of the elements and the parameters of the RGB model and have the values of the coefficients of determination *R*^2^ = 0.837 for nitrogen, *R*^2^ = 0.703 for phosphorus, and *R*^2^ = 0.703 for potassium. Therefore, the accuracy of the discussed procedure is rather low. The authors point few limitations of their solution. Pictures received with a long stick from the camera must be taken at the right angle to avoid too large area pictures of the field. Photograph taken from a height is not free from the influence of sunlight, from the reflection of light from rice fields, or from the shadows of for example selfie stick itself. Photos should be taken in favourable weather conditions avoiding very cloudy weather or when it is scorching heat. Moreover, the obtained results may be influenced by the variability of genotypes and age of rice cultivation. The optimal age of the rice crops used in this approach is the plant in the tillering stage.

**Fig. 7:**
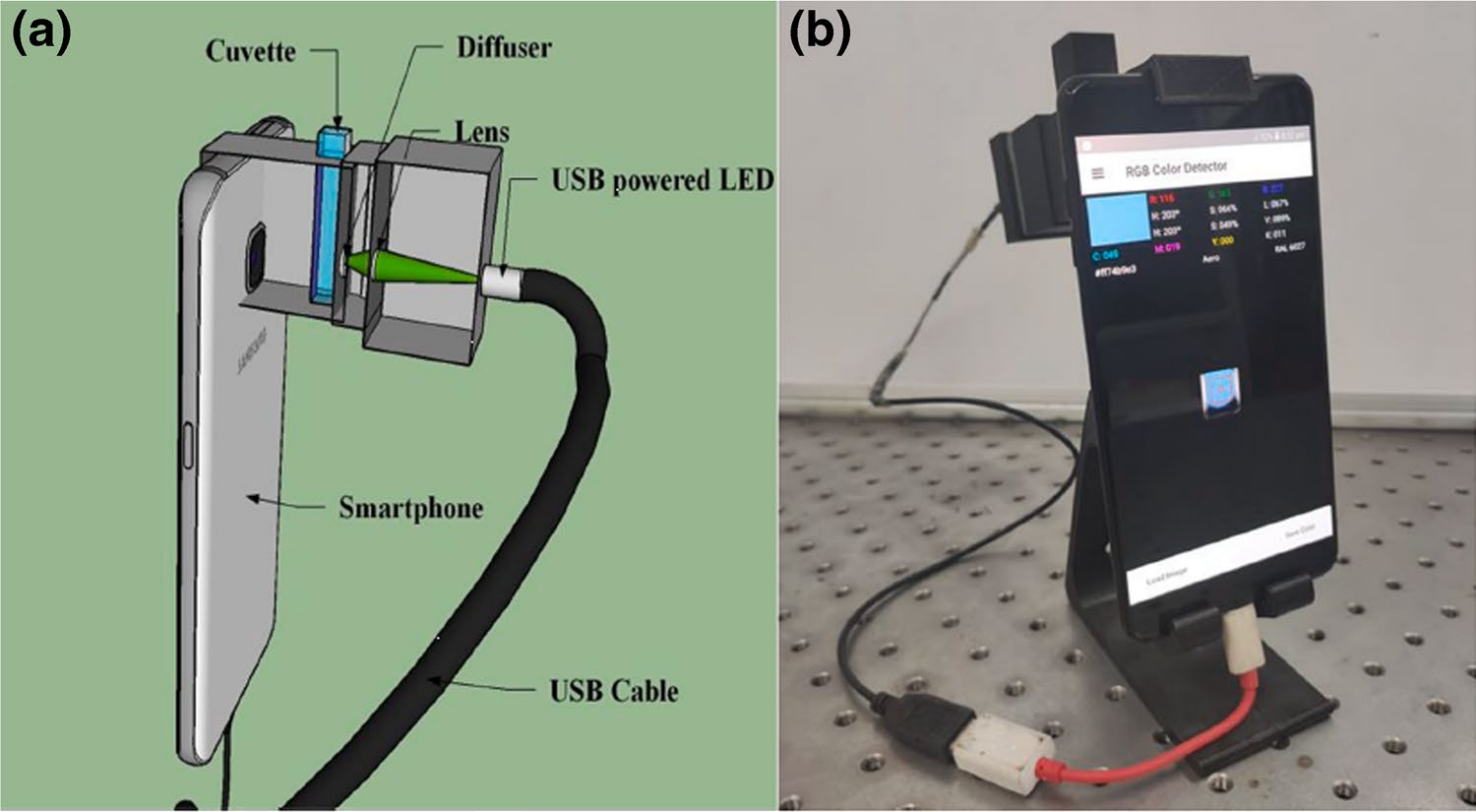
Set for measuring phosphate content in soil — diagram **a** and photo of the set **b**. Copied from Das, P., Chetry, B., Paul, S., Bhattacharya, S. S., & Nath, P. (2022). [38] Reprinted with permission from Elsevier

Another example of using a smartphone is the determination of the content of available phosphates in soil (the procedure can also be used to determine phosphate in water) is based on the analytes extraction with Bray II solution as with the standard procedure — the reaction gives a blue coloured product. Here, however, instead of using a molecular spectrophotometer, a smartphone was used with a connected LED lamp as an external source of radiation, as shown in Fig. 7 [38]. The set consists of a tripod in which a smartphone is placed and a place to put a litter box, lenses, and a light diffuser between the LED lamp and the camera. The results obtained with such a kit are very consistent with the results obtained with the laboratory procedure based on molecular spectrophotometry.

## Soil contamination monitoring

Soil contamination by naturally occurring and anthropogenic organic and inorganic chemicals is a serious human and environmental health problem. Soil contaminants include heavy metals, petroleum products, and organic xenobiotics such as pesticides, polycyclic aromatic hydrocarbons, and polychlorinated biphenyls. General problems associated with soil contamination monitoring are concerned with quantitative extraction of the desired analytes from the sample matrix.

### Inorganic contaminants monitoring

For the Pb, Cd, or Cr determination, a procedure has been applied based on gold nanoparticles with appropriate functional groups surface modification. The use of gold nanoparticles at the extraction stage is rather inconvenient in the case of field or home analyses; therefore, it is unlikely to find application in agriculture and will not be discussed further [22].

An analytical procedure was developed for the determination of As (III) in soil samples using a commercially available test strip with a smartphone colour reading [23]. The procedure involves extracting As from soil with water (the soluble fraction of As is determined) and then reducing As (III) to arsine (Reactions 1–2). It reacts with mercury bromide impregnated into the indicator paper (Reaction 3). The reaction results in a colour change to yellow–brown.

1 $${\mathrm{As}}_{2}{\mathrm{O}}_{3}+6\mathrm{ Zn}+12\mathrm{ HCl}\to 2\mathrm{ As}{\mathrm{H}}_{3}+6\mathrm{ ZnCl}2+3 {\mathrm{H}}_{2}\mathrm{O}$$

2 $${\mathrm{H}}_{3}{\mathrm{AsO}}_{4}+4\mathrm{ Zn}+8\mathrm{ HCl}\to {\mathrm{AsH}}_{3}+4\mathrm{ ZnC}{\mathrm{l}}_{2}+4 {\mathrm{H}}_{2}\mathrm{O}$$

3 $${\mathrm{AsH}}_{3}+{\mathrm{HgBr}}_{2}\to \mathrm{AsH}(\mathrm{HgBr}{)}_{2}+\mathrm{AsH}(\mathrm{HgBr}{)}_{3}$$

The content of As can be determined from concentration 0.005 mg L^−1^.

### Organic contaminants monitoring

Trinitrotoluene (TNT) may appear in the soil as a contaminant due to use of explosives. An analytical procedure was developed to determine this compound in soil with a smartphone as a detector [39]. The soil sampler was based on a piece of lignin-free linden wood. Then the surface of the wood was modified to contain chemically bonded amine groups. The determination of TNT content is based on the reaction of the analyte with the chemically bonded amino group to the wood surface. It is related to the formation of the Meisenheimer complex, which results in a change in the colour of the wood surface.

The soil TNT determination procedure is applicable to moist soil samples, sampling is based on capillary suction. The colour intensity of the modified wood is proportional to the concentration of TNT in the soil, which is the basis for smartphone determination. The detection limit of this methodology is 0.07 mg g^−1^ soil, which is not a very good result. Wooden sampler changes colour in contact with various concentrations of TNT in the soil, and the calibration curve.

A paper-based colorimetric sensing procedure coupled with smartphone has been developed for the pesticide exposure analysis in environmental samples including soil [40]. The imprinting metal–organic framework has been utilized for the sensitive and selective determination of thiacloprid at 0.01 μg g^−1^ level.

Another procedure base on smartphone-based image analysis has been proposed for detection of nonylphenol in soil. To improve selectivity, molecularly imprinted polymers/carbon dots coated on cotton fabrics were utilized. Due to high fluorescence emission, the received composite was used as a sensor for highly selective nonylphenol detection. The fluorescence images were taken by smartphone and analysed by software for RGB measurement [41].

Microplastics are ubiquitous in the environment and are among of the soil pollutants as well. Recently, smartphone-based method for rapid quantify determination of microplastics has been proposed. The method involves isolating of microplastics from soil or water by density separation and vacuum filtration, staining the isolated plastic polymers with the reagent Nile Red, and quantifying the strained microplastics as small as 10 µm using a smartphone-based fluorescence microscope with an opti-mechanical attachment. The authors note that the proposed method successfully detected a wide range of plastic polymers, but a dilution step was often needed if the samples contained high concentrations of particulates or non-plastic debris to minimize optical overlap or blocking [42].

The applications of smartphones in soil analysis are summarized in Table 1.

**Table 1:** Summary of smartphone-based imaging devices for soil quality control

Soil property	Parameter	Short description	Advantages	Reference
Contaminants content	Pesticides (thiacloprid)	Paper-based colorimetry coupled with further exploiting smartphone-assisted image acquisition and self-adaptive signal model	Point-of-use tracking of environmental pesticide expose and ecosystem protection in resource-deficient settings	[40]
	Nonylphenol	Utilization of molecularly imprinted polymers/carbon dots coated on cotton fabrics and smartphone-based image analysis with the use of RGB measurement	Highly selective procedure for nonylphenol determination in soil	[41]
	Microplastics	Smartphone-based fluorescence microscope with an opti-mechanical attachment	Method successfully detected a wide range of plastic polymers	[42]
	Nitrite ion	A carbon nanodots and neutral red-based photometric and fluorescence mode sensing with the use of handheld compact sensing platform developed on a smartphone	Low-cost, field portable, and relatively convenient to handle device for evolution of nitrite contamination	[43]
	Cr (VI)	Application carbon nanodots and RGB values of the images received for fluorescence efficiency which corresponds to concentration of Cr (VI)	Sensitive, convenient, and rapid detection devices for toxic Cr (VI) determination	[44]
	2,4,6-Trinitrotoluene	Modified wood-based chemical sensor for visual colorimetric detection. The photographs are collected by smartphone camera followed RGB analysis	Visual colorimetric sensor allows for simple, quick, sensitive, and selective detection of the analyte. Additionally, it is possible to directly distinguish the presence and semi-quantitative detection of the analyte by the naked eye	[39]
		A smart sensor with cellulose-based material was developed for the detection of 2,4,6-trinitrotoluene, using potassium hydroxide/ethanol reagent adsorbed onto a cellulose cotton swab. A red colour was observed immediately after exposure to the analyte	A simple and cheap method for 2,4,6-trinitrotoluene determination	[45]
	As (III)	Application of field test kits and smartphone-based optical sensing	Easy procedure for As determination	[46]
		Optical colorimetric and smartphone-integrated paper device for As (III) determination using sucrose modified gold nanoparticles as a nanoprobe	Smartphone-integrated paper based device is rapid and portable for monitoring of arsenic at the sample source	[47]
		Colorimetric detection protocol utilizing aptamers, gold nanoparticles, and NaCl coupled with smartphone detection	With the android application on the device to run the experiment, the whole process from sample preparation to detection is completed within 3 h without the necessity of skilled personnel	[48]
	Hg	Strategy for highly sensitive, selective, and colorimetric detection of mercury based on analyte-induced enhancement of the photocatalytic activity of TiO_2_–Au nanospheres toward degradation of methylene blue	A rapid, simple, sensitive, and specific detection approach was developed for quantitation of Hg^2+^ or CH_3_Hg^+^	[49]
	Fe	Developed platform consisted of two cuvettes, a digital imaging device, and white paper as a diffuser	Ease of accessibility, simplicity, sensitivity, and cost-effectiveness of our approach	[50]
Basic chemical and physical soil properties	Soil colour	Images taking by smartphone	Soil colour captured by smartphone image for soil profile horizon delineation	[51]
		Anew soil colour app for mobile phones. Various smartphone cameras were tested under sunny and cloudy conditions and compared with visual estimates using Munsell colour charts	Soil colour measured with smartphone cameras had less subjectivity and uncertainty	[23]
	Angularity	Images taking by smartphone with machine learning techniques application	Real-time angularity evaluations without demanding computations	[32]
	Soil texure	Cheap setup comprising a smartphone for predicting soil texture of the dried, ground, and sieved samples. The image acquisition system was used to capture triplicate images from 90 mineral soil samples, representing a wide textural variability from sand to clay	The rapid and noninvasive prediction of soil sand, silt, and clay	[52]
	pH	The sensing system consists of a 3D printed compact optical set-up which can be attached to the smartphone’s rear camera. The procedure involves measuring the change of transmission intensity, which is correlated with the change in the sample’s pH value	A cost-effective, compact, and handheld smartphone-based sensing tool for accurate estimation of pH values of agricultural farmlands	[30]
	Water content	Rapid and reasonable estimation of water content from a set of cellphone images	A proximal soil sensor to be used further for easy, rapid, and cost-effective analysis	[31]
Soil nutrients content	Phosphorus (in phosphate form)	Standard ascorbic acid protocol application where heteropoly acid-phosphomolybdic acid reagent changes colour upon treatment with ascorbic acid, which can be detected by the designed sensing system	With proposed sensing system, a common citizen can easily measure the phosphate concentration without the requirement of any laboratory equipment	[38]
	Soil organic matter	Soil organic matter content prediction based on smart phone photos and RGB colour distribution measurement	Good correlation between the colour parameters and soil organic matter was found	[35]
	Soil organic matter	Smartphone-based soil image segmentation technique and subsequent machine learning optimization methodology with a set of soil images for rapidly predicting soil organic matter	Soil organic matter prediction with significant time and cost savings	[33]
	pH, N-nitrate, available phosphorus (P), and exchangeable potassium (K)	A smartphone camera was used for the reading of the quick test colour	Easy performed quick test for soil nutrients content determination	[53]

## Outlook

Application of smartphones in agriculture is a promising tool to make a managing of the soil quality easy, user-friendly, and less time- and cost-consuming. On site analysis with the application of smartphones allows to receive results in real time condition directly in the sampling site.

Smartphones are generally used in three ways:

- To read colour more precisely than the human naked eye. The remaining of the analytical procedure is performed in quite traditional way,
- For colour reading, where the analytical procedure is modified in such a way that at the detection stage, it is possible to use a smartphone as a detector,
- The colour reading is an input to the neural network. Previously, it was trained to find relationships between the colour of a sample and the property or properties of soil samples.

The use of a smartphone is associated with the possibility of performing *on site* analysis, i.e. at the place of sampling, without the need to involve a chemical laboratory. Therefore, there is no need to transport samples, which may be associated with further cost reduction and limitation of the risk of losing the sample representativeness. However, the results of determinations using smartphones are quite uncertain, which means that they can be used as a rough evaluation tool.

Analysis of literature data of smartphones application for evaluation of soil quality shows that this is a relatively new promising area, due to most of the investigations were published in 2019–2022. However, smartphone-based procedures significantly differ both efforts made for its developments and the way how it could be applied. The easiest procedures assume application of test strips or paper-based colorimetry coupled with further exploiting smartphone-assisted image acquisition. Such procedures could be used for nutrient content analysis as well as contaminants determination. The great efforts were done to provide smartphone-based procedures for physical soil properties, such as soil colour analysis used for soil profiling. The reason is that such procedures require significant labor in the stage of the development and assume soil photos analysis and their correct interpretation for soil classification. Establish of the smartphone-based devices that could provide multicomponent analysis and integrates major soil parameters with the aim to overall soils quality seems to be the most perspective way of the modern agriculture development as well as smartphone integration into this area. Therefore, intensive research should be expected in this field which significantly contribute to the further expansion of the possibilities of smartphones application as an analytical tool.

*Limitation* of the smartphone’s application in the agriculture for chemical soil analysis could be connected to both chemical and technical reasons. Chemical limitations are related to the reaction used for the receiving of colour product for desired analytes and providing of colorimetric detection. The sensitivity of smartphone devices in many cases is considerably worse than for more sophisticated instruments like mass spectrometry; thus, smartphones are rarely used for trace analysis and determination of such soil pollutants as polycyclic aromatic hydrocarbons, pesticides, or antibiotics residue. Additionally, tedious sample preparation for trace analytes preconcentration and dealing with matrix effect is required, while smartphone-based device assumes fast and not labour intensive approaches. Technical limitation could be related to the smartphone itself, particularly in camera’s parameters. Different types of cameras from various manufacturers and with different lenses could be a reason of low reproducibility of the results on a large number of devices.

Among the *future trends*, all-in-one smartphone-based devices seem to be preferable for agricultural soils analysis that enables users to complete a self-assessment about soil quality and receive a performance report including actionable insight to identify how to improve soil quality for productive and sustainable agriculture.
